# Development of a pharmacological evidence‐based anticholinergic burden scale for medications commonly used in older adults

**DOI:** 10.1111/ggi.14619

**Published:** 2023-06-14

**Authors:** Shizuo Yamada, Masae Mochizuki, Junko Chimoto, Risa Futokoro, Satomi Kagota, Kazumasa Shinozuka

**Affiliations:** ^1^ Center for Pharma‐Food Research (CPFR), Graduate School of Pharmaceutical Sciences University of Shizuoka Shizuoka Japan; ^2^ School of Pharmacy and Pharmaceutical Sciences Mukogawa Women's University Nishinomiya Japan

**Keywords:** anticholinergic burden, Japan, medication lists, muscarinic receptors

## Abstract

**Aim:**

The present study aimed to develop a pharmacological evidence‐based anticholinergic burden scale (ABS) through a direct assessment of muscarinic receptor‐binding activities of 260 medications commonly used in older adults.

**Methods:**

The muscarinic receptor‐binding activities of 260 drugs were assessed by the displacement of specific [*N*‐methyl‐^3^H]scopolamine methyl chloride binding in the rat brain. The maximum blood concentrations (*C*_max_) of drugs after their administration to subjects were cited from their interview forms.

**Results:**

In total, 96 of 260 drugs displayed concentration‐dependent muscarinic receptor binding in rat brain. Based on muscarinic receptor‐binding activity (IC_50_) and *C*_max_ after the administration at clinical doses in humans, we rated ABS 3 (strong) for 33 drugs and ABS 2 (moderate) for 37 drugs. There was an approximate similarity between muscarinic receptor‐binding activities (IC_50_) and *C*_max_ of 33 drugs (ABS 3) after their administration at clinical doses in humans. Furthermore, 26 drugs were defined as ABS 1 (weak) by muscarinic receptor‐binding activity. The remaining 164 drugs exhibited slight or no significant muscarinic receptor‐binding activities at high concentration of 100 μM, and they were defined as ABS 0. There was a marked similarity for 28 drugs (ABS 3) between the present ABS data and their previous scoring data in the literature.

**Conclusions:**

To our knowledge, the present study developed the first comprehensive pharmacological evidence‐based ABS of drugs based on muscarinic receptor‐binding activity, which provides guidance as to which drugs may be discontinued to reduce anticholinergic burden. **Geriatr Gerontol Int 2023; 23: 558–564**.

## Graphical abstract

Drugs were defined as anticholinergic burden scale (ABS) 3 by their muscarinic receptor‐binding activity and maximum blood drug concentrations.In total, 96 of 260 drugs displayed concentration‐dependent muscarinic receptor binding in rat brain. Based on muscarinic receptor‐binding activity (IC_50_) and maximum blood concentration (*C*_max_) after the administration at clinical doses in humans, we rated ABS 3 (strong) for 33 drugs and ABS 2 (moderate) for 37 drugs. The present study developed the first comprehensive pharmacological evidence‐based ABS of drugs based on muscarinic receptor‐binding activity.

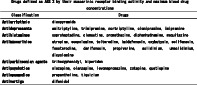

## Introduction

An increased life expectancy has been associated with better access to health care and treatment for many diseases. This has resulted in more elderly patients with multidrug regimens prescribed for multiple morbidities. Polypharmacy with anticholinergic agents is common in nursing home residents and markedly increases the risk of anticholinergic toxicity.^1^ More than 600 medicinal products with a broad therapeutic range and adverse effect profiles are recognized to exhibit anticholinergic activity,^2^, ^3^ and include psychoactive drugs, such as hypnotic, antipsychotic, antiparkinsonian, and antidepressant drugs, and non‐psychoactive drugs, such as cardiac, corticosteroid, and antibiotic drugs.^4^, ^5^ Drugs taken by patients may also exaggerate the anticholinergic effects of typical anticholinergics used frequently to treat overactive bladder and chronic obstructive pulmonary disease. Therefore, anticholinergic accumulation results in dry mouth, constipation, blurred vision, dry eyes, tachycardia, urinary retention, cognitive impairment, agitation, paranoia, anxiety, and delirium.^6^ Anticholinergic effects are caused by the significant blockade of muscarinic receptors in the parasympathetic and central nervous systems.^7^, ^8^ More receptors may be additively blocked when several agents are simultaneously administered, which is known as anticholinergic accumulation, and occurs because of polypharmacy and/or changed pharmacokinetics.^4^, ^9^, ^10^, ^11^ This accumulation may enhance anticholinergic adverse effects.

A rating scale to quantify the anticholinergic burden may be useful for reducing the risk of anticholinergic adverse events in elderly patients. Anticholinergic drug rating scales have been developed through collaborations between a number of investigators.^10^, ^12^, ^13^, ^14^, ^15^, ^16^, ^17^, ^18^, ^19^, ^20^, ^21^, ^22^, ^23^ In these scales, drugs are rated in an original manner from 0 to 3, with 0 signifying no known anticholinergic activity and 3 indicating marked anticholinergic activity. The scores of all medications are then summed to obtain the total score. The majority of previous studies evaluated the rating of anticholinergic scores using practitioners' knowledge of a list of drugs with known anticholinergic effects or serum anticholinergic activity,^23^, ^24^, ^25^, ^26^, ^27^ but not by the direct and quantitative assessment of the anticholinergic activity of individual drugs using pharmacological methods.

A reliable pharmacological assessment for clinically relevant anticholinergic activity involves directly elucidating whether drugs exhibit binding activity for muscarinic receptors and the functional antagonism of the cholinergic agonist‐induced response.^7^, ^8^ Furthermore, the findings obtained need to be confirmed by the presence of typical antimuscarinic adverse drug effects, such as dry mouth and constipation, in clinical studies. As an initial step, assessments of the presence or absence of muscarinic receptor‐binding activity are crucial for confirming the anticholinergic adverse event of each drug. A radioreceptor‐binding assay with a selective radioligand may be a simple and powerful pharmacological tool for evaluating the presence or absence of the anticholinergic activity for each drug.^8^

The population of Japan is aging rapidly, and although a number of drugs are clinically used in older adults, some are not included in previously developed scales, such as the anticholinergic cognitive burden.^19^, ^27^ Therefore, the present study aimed to develop a pharmacological evidence‐based anticholinergic burden scale (ABS) through direct assessments of the muscarinic receptor‐binding activities of medications commonly used in elderly Japanese.

## Methods

### Materials

[*N*‐methyl‐^3^H]scopolamine methyl chloride ([^3^H]NMS) (3.03–3.16 TBq/mmol) was purchased from PerkinElmer Life Sciences, Inc. All drugs and chemicals were purchased from commercial sources.

### Animals

Eight‐ to 10‐week‐old male Sprague–Dawley rats (250–300 g) were purchased from Japan SLC (Shizuoka, Japan). They were housed in the laboratory with free access to food and water and maintained on a 12‐h light/dark cycle in a room with a controlled temperature (24 ± 2°C).

Experimental protocols received approval from the Ethics Committee for Research at the University of Shizuoka (registration number: 136038), and were performed in accordance with the guidelines for the Care and Use of Laboratory Animals, which conformed to the provisions of the Declaration of Helsinki (as revised in Tokyo 2004).

### Muscarinic receptor‐binding assay

The muscarinic receptor‐binding assay was performed using [^3^H]NMS, a selective radioligand for muscarinic receptors, as previously described.^8^ Animals were exsanguinated by taking blood from the descending aorta under anesthesia with pentobarbital sodium (50 mg/kg, i.p.), and the whole brain, except for the cerebellum, was dissected. Brain tissue was carefully minced and homogenized using a Kinematica Polytron homogenizer in 19 volumes of ice‐cold 30 mM Na^+^/HEPES buffer (pH 7.5). The homogenate was then centrifuged at 40 000 *g* for 20 min. The resulting pellet was resuspended in the same buffer for the binding assay. In the competition experiment, the tissue homogenate was incubated with [^3^H]NMS (0.3 nM) in the presence of each drug. Incubations were performed at 25°C for 60 min and the reaction was terminated by rapid filtration (Cell Harvester; Brandel Co., Gaithersburg, MD, USA) through Whatman GF/B glass filters. Filters were rinsed three times with 3 mL of ice‐cold 50 mM Na^+^/K^+^ phosphate buffer (pH 7.4). Tissue‐bound radioactivity was extracted from the filters overnight in scintillation fluid, and radioactivity was measured using a liquid scintillation counter. Specific [^3^H]NMS binding was assessed experimentally from differences in counts between the absence and presence of 1 μM atropine. All steps were performed at 4°C to minimize the dissociation of drugs from receptor sites.

### Data analysis

The assessment of muscarinic receptor‐binding activity was based on the displacement of specific [^3^H]NMS binding in brain tissues by drugs. [^3^H]NMS binding data were subjected to a non‐linear regression analysis using GraphPad PRISM (ver. 5; GraphPad Software, San Diego, CA, USA). The ability of non‐labeled drugs to inhibit specific [^3^H]NMS binding (0.5 nM) was estimated from IC_50_, the molar concentration of drugs needed to displace 50% of specific [^3^H]NMS binding. The maximum blood concentration (*C*_max_) of drugs after their administration to subjects was cited from their interview forms. The interview form is “Prescription drug information forms” provided by pharmaceutical companies from Pharmaceuticals and Medical Devices Agency (PMDA) in Japan, https://www.pmda.go.jp. Data were presented as the means of four to seven experiments, with the standard error of mean (SEM) in footnotes of tables.

## Results

### Assessment of muscarinic receptor‐binding activity

A radioreceptor binding assay with [^3^H]NMS^8^ was used to assess the anticholinergic activities of 260 medications commonly used in elderly Japanese. The significant competitive inhibition of specific [^3^H]NMS binding in the rat brain was observed at a concentration of 100 μM by 96 of the 260 drugs examined. Based on these preliminary results, the concentration‐dependent displacement of specific [^3^H]NMS binding in the rat brain by five or six lower concentrations of 96 drugs was examined to estimate quantitatively their muscarinic receptor‐binding activity (IC_50_). These drugs displaced specific [^3^H]NMS binding at concentrations between 0.01 nM and 100 μM in a concentration‐dependent manner.

Specific [^3^H]NMS binding in the rat brain was displaced in a concentration‐dependent manner by lower concentrations (0.01–100 nM) of 31 drugs (Table 1), which included first‐generation antidepressants (amitriptyline, trimipramine), first‐generation antihistamines (cyproheptadine, clemastine, promethazine), second‐generation antihistamines (mequitazine, desloratadine), antimuscarinics (atropine, scopolamine, tolterodine, imidafenacin, oxybutynin, solifenacin, fesoterodine, darifenacin, aclidinium, umeclidinium, tiotropium, glycopyrronium, ipratropium, dicyclomine), antiparkinsonian agents (trihexyphenidyl, biperiden), antipsychotics (clozapine, olanzapine), antispasmodics (propantheline, tiquizium, butropium, mepenzolate), musculoskeletal agent (pridinol), and respiratory agent (cloperastine). These drugs displayed strong binding activity of muscarinic receptors at IC_50_ less than 100 nM for the competitive inhibition of specific [^3^H]NMS binding (Table 1).

**Table 1:** Pharmacological classification and muscarinic receptor‐binding activity (IC_50_) of drugs, which displayed IC_50_ values <100 nM

Classification	Drugs	IC_50_ (nM)	Classification	Drugs	IC_50_ (nM)
Antidepressants	Amitriptyline	29.3	Antimuscarinics	Aclidinium	0.049
(First generation)	Trimipramine	99.0	(Bronchodilator)	Umeclidinium	0.15
Antihistamines	Cyproheptadine	5.10		Tiotropium	0.39
(First generation)	Clemastine	20.4		Glycopyrronium	3.78
	Promethazine	42.3		Ipratropium	4.38
(Second generation)	Mequitazine	3.99	(Gastrointestinal agent)	Dicyclomine	<0.01
	Desloratadine	74.9	Antiparkinsonian agents	Trihexyphenidyl	10.4
Antimuscarinics	Atropine	3.41		Biperiden	10.5
	Scopolamine	1.00	Antipsychotics	Clozapine	70.4
(Urinary incontinence)	Tolterodine	1.22		Olanzapine	73.7
	Imidafenacin	3.66	Antispasmodics	Propantheline	0.12
	Oxybutynin	6.84		Tiquizium	1.96
	Solifenacin	7.38		Butropium	2.47
	Fesoterodine	12.0		Mepenzolate	3.06
	Darifenacin	18.0	Musculoskeletal agent	Pridinol	53.6
			Respiratory agent	Cloperastine	0.30

*Note*: IC_50_ (50% inhibitory potency of specific [^3^H]NMS binding): means of 4–7 experiments. Standard error of mean (SEM) for each IC_50_ value was less than 15% of mean value.

Furthermore, 39 drugs competed with specific [^3^H]NMS binding at IC_50_ ≥100 nM and <10 μM as shown in Table 2. These drugs displayed moderate binding activity of muscarinic receptors and included antiarrhythmic, anticholinesterase agent, antidementia agent, antidepressants, antidiabetic, antihistamines, antimuscarinic, antipsychotics, antispasmodics, antivertigo agent, β_3_‐adrenoceptor agonists, gastrointestinal agent, and respiratory agents. The 26 drugs displayed relatively weak binding activity of muscarinic receptors at IC_50_ ≥10 μM and <100 μM, as presented in Table 3, and included antiarrhythmics, antidepressant, antidiabetics, antihistamines, anti‐pollakisuria, antipsychotics, antithrombotics, cardiovascular agents, gastrointestinal agents, and respiratory agents. The remainder (164 drugs) of 260 drugs showed slight (<50%) or no inhibition of specific [^3^H]NMS binding at the concentration of 100 μM, and were defined as drugs with negligible or no receptor‐binding activity (Table S1).

**Table 2:** Pharmacological classification and muscarinic receptor‐binding activity (IC_50_) of drugs, which displayed IC_50_ values ≥100 nM and <10 μM

Classification	Drugs	IC_50_ (μM)	Classification	Drugs	IC_50_ (μM)
Antiarrhythmic	Disopyramide	5.99	Antimuscarinic	Propiverine	0.35
Anticholinesterase agent	Distigmine	1.70	Antipsychotics	Chlorpromazine	0.38
Antidementia agent	Donepezil	1.30		Levomepromazine	0.39
Antidepressants	Nortriptyline	0.17		Zotepine	0.55
(First generation)	Clomipramine	0.19		Quetiapine	1.52
	Imipramine	0.28		Prochlorperazine	4.28
	Amoxapine	1.12		Fluphenazine	6.52
(Second generation)	Maprotiline	1.32		Perphenazine	7.00
	Mianserin	1.42	Antispasmodics	Scopolamine N‐butyl	0.30
	Setiptiline	1.97		Eperisone	0.90
(Third generation)	Paroxetine	0.55	Antivertigo agent	Difenidol	0.33
	Sertraline	6.12	β_3_‐Adrenoceptor agonists	Vibegron	5.32
(Fourth generation)	Mirtazapine	1.53		Mirabegron	6.62
(Selective NRI)	Reboxetine	7.37	Gastrointestinal agent	Vonoprazan	1.70
Antidiabetic	Linagliptin	2.89	Respiratory agents	Procaterol	3.90
Antihistamines	Dimenhydrinate	0.45		Salmeterol	6.10
(First generation)	Diphenhydramine	0.50		Vilanterol	6.10
	Chlorpheniramine	5.10		Fluticasone Propionate	8.00
	Hydroxyzine	9.12			
(Second generation)	Ebastine	0.76			
	Rupatadine	1.51			

*Note*: IC_50_ (50% inhibitory potency of specific [^3^H]NMS binding): means of 4–7 experiments. Standard error of mean (SEM) for each IC_50_ value was less than 20% of mean value.

**Table 3:** Pharmacological classification and muscarinic receptor‐binding activity (IC_50_) of drugs, which displayed IC_50_ values ≥10 μM and <100 μM, and they were defined as anticholinergic burden scale 1

Classification	Drugs	IC_50_ (μM)	Classification	Drugs	IC_50_ (μM)
Antiarrhythmics	Cibenzoline	20.1	Antithrombotics	Ticlopidine	18.0
	Bepridil	20.8		Limaprost	56.1
	Quinidine	44.6	Cardiovascular agents	Doxazosin	13.2
Antidepressant	Duloxetine	19.3		Verapamil	23.7
Antidiabetics	Saxagliptin	19.1		Amlodipine	99.1
	Empagliflozin	22.1	Gastrointestinal agents	Domperidone	15.6
	Alogliptin	81.5		Rabeprazole	18.1
Antihistamines	Olopatadine	25.1		Loperamide	31.8
(Second generation)	Epinastine	26.3		Acotiamide	44.2
	Loratadine	81.1	Respiratory agents	Formoterol	14.3
Anti‐pollakisuria	Flavoxate	11.5		Fluticasone furoate	18.2
Antipsychotics	Haloperidol	16.7		Indacaterol	39.3
	Risperidone	21.5		Tulobuterol	71.9

*Note*: IC_50_ (50% inhibitory potency of specific [^3^H]NMS binding): means of 4–7 experiments. Standard error of mean (SEM) for each IC_50_ value was less than 20% of mean value.

### Definition of anticholinergic burden scale in consideration of  blood concentrations of drugs in subjects after their administration at clinical doses

The evaluation of muscarinic receptor‐binding activity under *in vitro* conditions may be limited in part because the drug concentrations tested may not reflect those in biological fluids under clinical conditions. To assess the clinical significance of the ABS rating based on muscarinic receptor‐binding activity, we compared the IC_50_ (Tables 1 and 2) of specific [^3^H]NMS binding with *C*_max_ in subjects after the administration of each drug at clinical doses. *C*_max_ was cited from the interview form of each drug as described in the Methods section. As shown in Table 4, IC_50_ values for the majority of drugs were ≤C_max_, while IC_50_ values for 6 drugs (nortriptyline, clomipramine, diphenhydramine, propiverine, levomepromazine, difenidol) were greater than *C*_max_; however, this difference was less than three‐fold. Generally, comparisons showed an approximate similarity between muscarinic receptor‐binding activity (IC_50_ of inhibition of specific [^3^H]NMS binding) and *C*_max_ for 33 drugs after their administration at clinical doses. Therefore, we defined these drugs with IC_50_ ≤*C*_max_ or within three‐fold of *C*_max_ as ABS 3, in consideration of their blood concentrations in subjects after their administration at clinical doses (Table 4). Notably, IC_50_ for 10 drugs (disopyramide, nortriptyline, clomipramine, imipramine, diphenhydramine, propiverine, levomepromazine, zotepine, quetiapine, and difenidol) in Table 2, were approximately similar to their *C*_max_. Therefore, we included these drugs as ABS 3 in consideration of *C*_max_ (Table 4). Furthermore, as shown in Table 5, 37 drugs with three‐fold greater IC_50_ than *C*_max_ were rated as ABS 2. These included 8 drugs (desloratadine, tiotropium, glycopyrronium, ipratropium, butropium, mepenzolate, pridinol, and cloperastine) presented in Table 1, and 29 drugs presented in Table 2. Furthermore, 26 drugs presented in Table 3 were defined as ABS 1. The remainder (164 drugs) of 260 drugs, which displayed slight (<50%) or no inhibition of specific [^3^H]NMS binding at 100 μM were defined as drugs with ABS 0 (negligible or no binding activity of muscarinic receptor) (Table S1).

**Table 4:** Drugs defined as anticholinergic burden scale 3 with their pharmacological classification and muscarinic receptor‐binding activity (mean IC_50_) in consideration of maximum blood drug concentrations (C_max_) and clinical doses

Classification	Drugs	IC_50_ (nM)	C_max_ (nM)	Dose [repeated admin.]
Antiarrhythmic	Disopyramide	5990	4390	[100 mg × 3/day]
Antidepressants (First generation)	Amitriptyline	29.3	284.8	[125 ~ 180 mg/day]
	Trimipramine	99.0	95.8	[50 mg]*
	Nortriptyline	168	69.5	[25 mg]*
	Clomipramine	188	87.7	[50 mg]*
	Imipramine	279	250	[75 mg/day]
Antihistamines (First generation)	Cyproheptadine	5.10	174.0	[5 mg]*
	Clemastine	20.4	42.0	[2 mg]*
	Promethazine	42.3	137.8	[50 mg]*
	Diphenhydramine	498	260	[50 mg]*
(Second generation)	Mequitazine	3.99	66.5	[6 mg × 2/day]
Antimuscarinics	Atropine	3.41	25.9	[0.02 mg/kg]i.m.*
	Scopolamine	1.00	29.6	[0.6 mg]s.c.*
(Urinary incontinence)	Tolterodine	1.22	3.99	[4 mg × 1/day]
	Imidafenacin	3.66	3.88	[0.25 mg × 2/day]
	Oxybutynin	6.84	18.7	[3 mg]*
	Solifenacin	7.38	116.3	[10 mg × 1/day]
	Fesoterodine	12.0	5‐HMT 11.0	[8 mg × 1/day]
	Darifenacin	18.0		Not approved in Japan
	Propiverine	350	153	[20 mg]*
(Bronchodilator)	Aclidinium	0.049	0.397	[400 μg × 2/day] inhale
	Umeclidinium	0.15	0.660	[125 μg × 1/day] inhale
(Gastrointestinal agent)	Dicyclomine	< 0.01	62.6	[10 mg]*
Antiparkinsonian agents	Trihexyphenidyl	10.4	166.0	[8 mg]*
	Biperiden	10.5	16.4	[4 mg]*
Antipsychotics	Clozapine	70.4	3488.1	[150 mg × 2/day]
	Olanzapine	73.7	89.9	[10 mg × 1/day]
	Levomepromazine	394	150	[100 mg]*
	Zotepine	554	389	[100 mg]*
	Quetiapine	1520	1260	[100 mg × 2/day]
Antispasmodics	Propantheline	0.12	118.4	[60 mg]*
	Tiquizium	1.96	33.5	[10 mg]*
Antivertigo	Difenidol	325	191	[25 mg]*

*Note*: IC_50_ (50% inhibitory potency of specific [^3^H]NMS binding): means of 4–7 experiments.

* Single administration. 5‐HMT, 5‐hydroxymethyltolterodine.

**Table 5:** Drugs defined as anticholinergic burden scale 2 with their pharmacological classification and muscarinic receptor‐binding activity (mean IC_50_) in consideration of maximum blood drug concentrations (C_max_) and clinical doses

Classification	Drugs	IC_50_ (μM)	Cmax (μM)	Dose [repeated admin.]
Antidementia agent	Donepezil	1.30	0.0690	[5 mg × 1/day]
Anticholinesterase agent	Distigmine	1.70	0.00763	[5 mg]*
Antidepressants				
(First generation)	Amoxapine	1.12	0.149	[50 mg]*
(Second generation)	Maprotiline	1.32	0.277	[25 mg × 3/day]
	Mianserin	1.42	0.128	[60 mg × 1/day]
	Setiptiline	1.97	0.00371	[1 mg]*
(Third generation)	Paroxetine	0.545	0.181	[20 mg × 1/day]
	Sertraline	6.12	0.228	[100 mg × 1/day]
(Fourth generation)	Mirtazapine	1.53	0.314	[30 mg × 1/day]
(Selective NRI)	Reboxetine	7.37	Not approved in Japan	
Antidiabetic	Linagliptin	2.89	0.0254	[5 mg × 1/day]
Antihistamines (First generation)	Dimenhydrinate	0.446	0.102	[50 mg]*
	Chlorpheniramine	5.10	0.0328	[4 mg]*
	Hydroxyzine	9.12	0.193	[0.7 mg/kg]*
(Second generation)	Desloratadine	0.0749	0.0135	[5 mg × 1/day]
	Ebastine	0.755	^carebastine^ 0.208	[10 mg]*
	Rupatadine	1.51	0.0256	[20 mg × 1/day]
Antimuscarinics (Bronchodilator)	Tiotropium	0.00039	0.0000131	[5 μg × 1/day] inhale
	Glycopyrronium	0.00378	0.000521	[50 μg × 1/day] inhale
	Ipratropium	0.00438	Not reported	
Antipsychotics	Chlorpromazine	0.376	0.0336	[50 mg]*
	Prochlorperazine	4.28	Not reported	
	Fluphenazine	6.52	Not reported	
	Perphenazine	7.00	0.0005 ~ 0.012	[2 ~ 24 mg/man]*
Antispasmodics	Butropium	0.00247	Not reported	
	Mepenzolate	0.00306	Not reported	
	Scopolamine N‐butyl	0.304	Not reported	
	Eperisone	0.902	0.0305	[150 mg × 1/day]
β_3_‐Adrenoceptor agonists (Urinary incontinence)	Vibegron	5.32	0.354	[100 mg × 1/day]
	Mirabegron	6.62	0.343	[100 mg × 1/day]
Gastrointestinal agent	Vonoprazan	1.70	0.0675	[20 mg × 1/day]
Musculoskeletal agent	Pridinol	0.0536	Not reported	
Respiratory agents	Cloperastine	0.0003	Not reported	
	Procaterol	3.90	0.000470	[50 μg]*
	Salmeterol	6.10	0.00109	[200 μg] inhale*
	Vilanterol	6.10	0.000638	[25 μg × 1/day] inhale
	Fluticasone propionate	8.00	0.000719	[400 μg × 2/day] inhale

*Note*: IC_50_ (50% inhibitory potency of specific [^3^H]NMS binding): means of 4–7 experiments.

* Single administration.

We compared muscarinic receptor‐binding activity‐based ABS of 33 drugs rated as ABS 3 (Table 4) with their previously reported scale rating in 12 studies.^10^, ^12^, ^13^, ^14^, ^15^, ^16^, ^17^, ^18^, ^19^, ^20^, ^21^, ^22^ As shown in Table S2, there were marked similarities for 28 drugs between the present ABS data (Table 4) and previous scoring data in literatures, except for disopyramide, olanzapine, and quetiapine, which showed large variations (0–3) among studies. The scale of ABS 3 for aclidinium and umeclidinium (bronchodilators) was the first report.

## Discussion

In the present study, the muscarinic receptor‐binding activities of 260 drugs that are commonly used in elderly Japanese were assessed by the displacement of specific [^3^H]NMS binding in the rat brain.^8^ In total, 96 drugs exhibited muscarinic receptor‐binding activity in concentration‐dependent manner. We defined the ABS rating of drugs, based on the muscarinic receptor‐binding activity (IC_50_) and blood concentration (*C*_max_) after the administration at clinical doses in humans (Tables 4 and 5), and on quantitative assessments of muscarinic receptor‐binding activity (Tables 1, 2, 3, S1). To our knowledge, the present study has developed the first comprehensive assessment of the anticholinergic activity of each medication by pharmacological methods.

Previous studies evaluated the rating of anticholinergic scores using practitioners' knowledge of a list of drugs with known anticholinergic effects or serum anticholinergic activity.^10^, ^12^, ^13^, ^14^, ^15^, ^16^, ^17^, ^18^, ^19^, ^20^, ^21^, ^22^, ^23^, ^26^, ^27^ Marked variabilities and inconsistencies exist among these anticholinergic scales, including the number and potency score of anticholinergic medication.^23^, ^28^, ^29^ The use of serum anticholinergic activity has limitations, such as the inability to assess the anticholinergic effects of each individual drug or the potentially distorting effects of endogenous serum proteins.^26^ In previous studies, serum anticholinergic activity was not associated with anticholinergic adverse drug events^24^ or four anticholinergic drug scoring systems.^25^ None of the previous scales have comprehensively assessed the presence or absence of anticholinergic effects by each medication through pharmacological methods.

We herein demonstrated that 96 of 260 drugs displayed concentration‐dependent muscarinic receptor binding in the rat brain. Based on the muscarinic receptor‐binding activities (IC_50_) of 96 drugs, we found 31 drugs with strong binding activity of muscarinic receptors exerting IC_50_ less than 100 nM, 39 drugs with moderate binding activity exerting IC_50_ ≥100 nM and <10 μM, and 26 drugs with weak binding activity exerting IC_50_ ≥10 μM and <100 μM (Tables 1, 2, 3). All drugs in Table 1 concentration‐dependently inhibited the cholinergic agonist‐evoked contractions of rat ileal smooth muscle in an organ bath assay, indicating their cholinergic antagonistic effects (our unpublished data).

The *in vitro* muscarinic receptor‐binding activities of drugs may be limited in part because the concentrations tested may not reflect those in the human body under clinical conditions. Considerations of the anticholinergic potency and clinical dose of each drug may be required for the accurate quantification of the anticholinergic burden in assessments of the relative risks versus benefits of prescribing drugs. Therefore, we compared the muscarinic receptor‐binding activity (IC_50_) of each drug, which was presented in Tables 1, 2, with its therapeutic blood concentration (*C*_max_) following clinical dosing in humans. Based on the close approximation of IC_50_ and *C*_max_, 33 drugs were defined as drugs with ABS 3 as shown in Table 4, which included 10 drugs in Table 2, in addition to 23 drugs in Table 1. Furthermore, 37 drugs with three‐fold greater IC_50_ than *C*_max_ were finally rated as ABS 2 (Table 5), and 26 drugs presented in Table 3 were defined as ABS 1. The remainder (164 drugs), which displayed slight or no inhibition of specific [^3^H]NMS binding at 100 μM, were defined as drugs with ABS 0 (negligible or no binding activity of muscarinic receptor) (Table S1).

The present muscarinic receptor‐binding activity‐based ABS of 33 drugs rated as ABS 3 was compared with their previously reported scale rating in 12 studies (Table S2).^10^, ^12^, ^13^, ^14^, ^15^, ^16^, ^17^, ^18^, ^19^, ^20^, ^21^, ^22^ These scales assign scores to anticholinergic medications, with lower scores (typically 0–1) indicating no or the limited anticholinergic activity of a medication, intermediate scores (generally 2) indicating moderate anticholinergic activity, and high scores (usually 3) indicating strong anticholinergic activity. A cross‐sectional study to validate the anticholinergic risk scale in 249 participants showed that higher anticholinergic risk scale scores were associated with greater numbers of anticholinergic adverse events, including confusion, falls, dry mouth, dry eyes, and constipation.^15^ As shown in Table S2, there were marked similarities for 28 drugs between the present ABS data (Table 4) and previous scoring data in the literature. This result confirmed that drugs with clinically established anticholinergic effects in previous studies exhibited high affinities for muscarinic receptors.

A limitation of our newly developed ABS is that the anticholinergic activities of active metabolites that formed after the oral administration of drugs were not evaluated. In this case, the summation of the unchanged form and active metabolites needs to be considered. The muscarinic receptor‐binding activities of drugs depend on drug concentrations in tissues rather than plasma concentrations. Therefore, pharmacokinetic factors, such as the tissue concentrations of drugs, need to be considered for the precise assessment of anticholinergic activity because some drugs accumulate within tissues through sustained binding to tissue proteins by their repeated administration, which may indicate a markedly higher drug concentration in tissues than in plasma. In older adults, the renal excretion and hepatic metabolism of drugs are reduced, which may allow drugs to accumulate in tissues, indicating enhanced anticholinergic effects.^30^ An increasing age may also make the blood–brain barrier more permeable, which may be an issue because 70% of patients treated for overactive bladder with anticholinergics are 61–80 years old.^3^ Much caution should be paid for the central adverse effects in case that drugs with ABS 3 and 2 drugs are prescribed in patients. Therefore, the accuracy of the ABS rating may be increased by considering tissue (brain etc.) drug concentrations after drug administration.

In conclusion, this newly developed pharmacological evidence‐based burden scale has the potential as a practical tool for assessing the anticholinergic burden, specifically for risk reductions of anticholinergic adverse events in the poly‐medicated elderly.

## Author contributions

All authors contributed to the development of the manuscript, approved the final draft of the manuscript before its submission, and agreed to be accountable for all aspects of the work. SY, SK, and KS contributed to the conception and design of the study; SY, JC, MM, and RF acquired the data; SY, JC, MM, and SK conducted the data analysis and interpretation; SY obtained funding; SY, JC, SK, and KS provided administrative, technical, or material support; SY, SK, and KS provided supervision; SY, MM, SK, and KS contributed to the preparation, review, and finalization of the study. The interpretation and conclusions contained in the present study are those of the authors alone.

## Funding information

This study was financially supported in part by a Faculty Special Research Grant from the University of Shizuoka.

## Disclosure statement

All authors declare that they have no conflicts of interest.

## Supporting information


**Table S1.** Drugs defined as anticholinergic burden scale (ABS) 0 with their pharmacological classification by slight or no inhibition of specific [^3^H]NMS binding at the concentration of 100 μM.

**Table S2.** Comparison of anticholinergic burden rating scales of 33 drugs (defined as ABS 3 in Table 4) between the present study and previous studies (Ref. 10, 12–22)
Carnahan *et al*.,^12^ Ancelin *et al*.,^13^ Chew *et al*.,^14^ Rudolph *et al*.,^15^ Han *et al*.,^16^ Ehrt *et al*.,^17^ Sittironnarit *et al*.,^18^ ACB Scale 2012,^19^ Kalisch Ellett *et al*.,^10^ Salahudeen *et al*.,^20^ The 2019 AGS Beers Criteria,^21^ Jun *et al*.^22^ H, high; L, low; M, moderate.

## Data Availability

The data that support the findings of this study are available from the corresponding author upon reasonable request.
