# Supplementary material for: Development of a pharmacological evidence‐based anticholinergic burden scale for medications commonly used in older adults
Source: Geriatr Gerontol Int. 2023 Jun 14;23(7):558–64. doi: 10.1111/ggi.14619 (PMC11503540; doi:10.1111/ggi.14619)
Supplement: Supplementary file 1 — Table S1. Drugs defined as anticholinergic burden scale (ABS) 0 with their pharmacological classification by slight or no inhibition of specific [3H]NMS binding at the concentration of 100 μM. [file GGI-23-558-s002.docx]

Table S1. Drugs defined as anticholinergic burden scale (ABS) 0 with their pharmacological classification by slight or no inhibition of specific [^3^H]NMS binding at the concentration of 100 μM.

| Classification | Drugs |
| --- | --- |
| **Analgesics** | acetaminophen, aspirin, celecoxib, diclofenac, etodolac, lornoxicam, loxoprofen, pregabalin, tramadol, zaltoprofen |
| **Antiarrhythmics** | atenolol, bisoprolol, flecainide, mexiletine, pilsicainide, propranolol |
| **Antidementia agents** | galantamine, memantine, rivastigmine |
| **Antidepressants** | fluvoxamine, milnacipran, trazodone, venlafaxine |
| **Antidiabetics** | anagliptin, canagliflozin, dapagliflozin, epalrestat, glimepiride, ipragliflozin, metformin, miglitol, pioglitazone, sitagliptin, teneligliptin, tofogliflozin, voglibose, vildagliptin |
| **Antiepileptics** | carbamazepine, valproate-Na |
| **Antihistamines** | bepotastine, bilastine, cetirizine, fexofenadine, levocetirizine, meclizine |
| **Antihyperlipidemics** | atorvastatin, bezafibrate, ethyl-docosahexaenoate, ethyl-icosapentaenoate, ezetimibe, fenofibrate, fluvastatin, pitavastatin, pravastatin, rosuvastatin, simvastatin |
| **Anti-infectives** | clarithromycin, levofloxacin |
| **Antiparkinsonian agents** | amantadine, levodopa, pramipexole |
| **Antipsychotics** | aripiprazole, pimozide, sulpiride |
| **Antithrombotics** | apixaban, cilostazol, clopidogrel, dabigatran, edoxaban, rivaroxaban, warfarin |
| **Antivertigo** | betahistine |
| **Anxiolytics, Hypnotics, sedatives** | alprazolam, bromazepam, brotizolam, chlordiazepoxide, clobazam, clonazepam, clorazepate, diazepam, estazolam, etizolam, flunitrazepam, flurazepam, ethyl-loflazepate, lorazepam, lormetazepam, medazepam, midazolam, nimetazepam, nitrazepam, oxazepam quazepam, ramelteon, tandospirone, temazepam, tofisopam, triazolam, zaleplon, zolpidem, zopiclone |
| **Cardiovascular agents** | azilsartan, benidipine, candesartan, carvedilol, cilnidipine, digoxin, diltiazem, irbesartan, isosorbide-dinitrate, β-methyl-digoxin, midodrine, nicorandil, nifedipine, olmesartan, telmisartan, valsartan |
| **Diuretics** | azosemide, furosemide, hydrochlorothiazide, spironolactone, torasemide, trichlormethiazide |
| **Gastrointestinal agents** | cimetidine, esomeprazole, famotidine, lansoprazole, metoclopramide, mosapride, omeprazole, ranitidine, rebamipide, sennoside, teprenone, ursodeoxycholic-acid |
| **Gout suppressants** | allopurinol, benzbromarone, colchicine, febuxostat, probenecid, potassium-citrate,  sodium-citrate, topiroxostat |
| **Hormonal preparations** | hydrocortisone, levothyroxine, prednisolone |
| **Minerals and vitamins** | magnesium-oxide, mecobalamin |
| **Musculoskeletal agents** | baclofen, methocarbamol, tizanidine |
| **Prostatic hypertrophy agents** | dutasteride, naftopidil, silodosin, tadalafil, tamsulosin, urapidil |
| **Respiratory agents** | ambroxol, budesonide, diprophylline, montelukast, pranlukast, salbutamol, theophylline |
