# Supplementary material for: Development of a pharmacological evidence‐based anticholinergic burden scale for medications commonly used in older adults
Source: Geriatr Gerontol Int. 2023 Jun 14;23(7):558–64. doi: 10.1111/ggi.14619 (PMC11503540; doi:10.1111/ggi.14619)
Supplement: Supplementary file 2 — Table S2. Comparison of anticholinergic burden rating scales of 33 drugs (defined as ABS 3 in Table 4) between the present study and previous studies (Ref. 10, 12–22) Carnahan et al.,12 Ancelin et al.,13 Chew et al.,14 Rudolph et al.,15 Han et al.,16 Ehrt et al.,17 Sittironnarit et al.,18 ACB Scale 2012,19 Kalisch Ellett et al.,10 Salahudeen et al.,20 The 2019 AGS Beers Criteria,21 Jun et al.22 H, high; L, low; M, moderate. [file GGI-23-558-s001.docx]

Table S2. Comparison of anticholinergic burden rating scales of 33 drugs (defined as ABS 3 in Table 4) between the present study and previous studies (References: 10, 12-22)

| Ref  Drugs | ABS | 12 | 13 | 14 | 15 | 16 | 17 | 18 | 19 | 10 | 20 | 21 | 22 |
| --- | --- | --- | --- | --- | --- | --- | --- | --- | --- | --- | --- | --- | --- |
| Aclidinium | 3 |  |  |  |  |  |  |  |  |  |  |  |  |
| Amitriptyline | 3 | 3 | 3 | 3 | 3 | 3 | 3 | 3 | 3 | 3 | H | Strong | 3 |
| Atropine | 3 | 3 |  | 3 | 3 | 3 |  | 3 | 3 |  | H | Strong | 3 |
| Biperiden | 3 |  |  |  |  |  |  |  |  | 3 |  |  | 3 |
| Clemastine | 3 | 3 |  |  |  |  |  |  | 3 |  | H | Strong | 3 |
| Clomipramine | 3 | 3 | 3 |  |  |  |  |  | 3 | 3 | H | Strong | 3 |
| Clozapine | 3 | 3 |  | 3 | 2 |  | 3 |  | 3 |  | H，M | Strong | 3 |
| Cyproheptadine | 3 | 2 |  |  | 3 |  |  | 3 | 2 | 3 | H，M | Strong | 2 |
| Darifenacin | 3 | 3 |  |  |  |  |  |  | 3 |  | H | Strong |  |
| Dicyclomine | 3 | 3 |  | 3 | 3 |  |  |  | 3 |  | H | Strong | 3 |
| Difenidol | 3 |  |  |  |  |  |  |  |  |  |  |  | 2 |
| Diphenhydramine | 3 | 3 |  | 2 | 3 | 3 |  |  | 3 |  | H | Strong | 3 |
| Disopyramide | 3 | 2 |  |  |  |  | 0 | 0 | 1 | 2 | M，L | Strong |  |
| Fesoterodine | 3 |  |  |  |  |  |  |  | 3 |  | H | Strong | 3 |
| Imidafenacin | 3 |  |  |  |  |  |  |  |  |  |  |  | 3 |
| Imipramine | 3 | 3 | 3 |  | 3 | 3 |  | 3 | 3 | 3 | H | Strong | 3 |
| Levomepromazine | 3 | 2 | 3 |  |  |  |  |  |  |  | H |  | 2 |
| Mequitazine | 3 |  |  |  |  |  |  |  |  |  |  |  | 3 |
| Nortriptyline | 3 | 3 |  | 2 | 2 | 3 | 2 |  | 3 | 3 | H，M | Strong | 3 |
| Olanzapine | 3 | 1 |  | 2 | 2 | 1 | 2 |  | 3 | 3 | H，M，L | Strong | 3 |
| Oxybutynin | 3 | 3 | 3 | 2 | 3 |  | 3 | 2 | 3 | 3 | H，M | Strong | 3 |
| Promethazine | 3 | 3 |  |  | 3 |  |  | 0 | 3 | 3 | H | Strong |  |
| Propantheline | 3 | 3 |  |  |  | 2 |  |  | 3 | 3 | H，M | Strong |  |
| Propiverine | 3 |  |  |  |  |  |  |  | 3 |  | H |  | 3 |
| Quetiapine | 3 | 0 |  | 1 | 1 | 2 | 1 |  | 3 | 2 | H，M，L |  | 2 |
| Scopolamine | 3 | 3 |  |  |  | 3 |  |  | 3 |  | H | Strong | 3 |
| Solifenacin | 3 |  |  |  |  |  |  |  | 3 |  | H | Strong | 3 |
| Tiquizium | 3 |  |  |  |  |  |  |  |  |  | H |  | 3 |
| Tolterodine | 3 | 3 |  | 3 | 2 | 3 |  | 3 | 3 |  | H，M | Strong | 3 |
| Trihexyphenidyl | 3 | 3 | 3 |  |  | 3 | 3 |  | 3 | 3 | H | Strong | 3 |
| Trimipramine | 3 | 3 | 3 |  |  |  | 3 |  | 3 |  | H | Strong |  |
| Umeclidinium | 3 |  |  |  |  |  |  |  |  |  |  |  |  |
| Zotepine | 3 |  |  |  |  |  |  |  |  |  |  |  | 2 |

Carnahan et al. [12], Ancelin et al. [13], Chew et al. [14], Rudolph et al. [15], Han et al. [16], Ehrt et al. [17], Sittironnarit et al. [18], ACB Scale 2012 [19], Kalisch Ellett et al. [10], Salahudeen et al. [20], The 2019 AGS Beers Criteria [21], Jun et al. [22]. H: high, M: moderate, L: low.
